# Varicella-Zoster Virus in Perth, Western Australia: Seasonality and Reactivation

**DOI:** 10.1371/journal.pone.0151319

**Published:** 2016-03-10

**Authors:** Igor A. Korostil, David G. Regan

**Affiliations:** The Kirby Institute, UNSW Australia, Sydney, New South Wales, Australia; University of Cincinnati School of Medicine, UNITED STATES

## Abstract

**Background:**

Identification of the factors affecting reactivation of varicella-zoster virus (VZV) largely remains an open question. Exposure to solar ultra violet (UV) radiation is speculated to facilitate reactivation. Should the role of UV in reactivation be significant, VZV reactivation patterns would generally be expected to be synchronous with seasonal UV profiles in temperate climates.

**Methods:**

We analysed age and gender specific VZV notification time series data from Perth, Western Australia (WA). This city has more daily sunshine hours than any other major Australian city. Using the cosinor and generalized linear models, we tested these data for seasonality and correlation with UV and temperature.

**Results:**

We established significant seasonality of varicella notifications and showed that while herpes-zoster (HZ) was not seasonal it had a more stable seasonal component in males over 60 than in any other subpopulation tested. We also detected significant association between HZ notifications and UV for the entire Perth population as well as for females and males separately. In most cases, temperature proved to be a significant factor as well.

**Conclusions:**

Our findings suggest that UV radiation may be important for VZV reactivation, under the assumption that notification data represent an acceptably accurate qualitative measure of true VZV incidence.

## Introduction

Varicella-zoster virus (VZV) infection often observed in children is known as varicella or chickenpox. This disease usually lasts a short time and its main symptom is a rash. However, VZV remains dormant in the nervous system and can reactivate causing a vesicular rash known as herpes zoster (HZ) or shingles [1]. The latter usually happens in older adults.

Current understanding of VZV epidemiology is not comprehensive and interpretation of some of its aspects remains limited to speculations based on inconsistent evidence. One example is the apparent dependence of VZV epidemiology on climate. As tropical and temperate climatic zones substantially differ in the amount and extent of variation of UV radiation (henceforth UV) they receive, a recently proposed hypothesis is that UV is likely to be a factor in determining the differences in global epidemiology of VZV [2–4]. It is possible that VZV can be deactivated by UV which would explain increased (decreased) incidence of varicella in temperate climates during the periods characterised by lower (higher) UV [3]. On the other hand, suppression of cellular immunity by UV [2] was proposed as a potential biological factor driving reactivation of VZV as HZ [5]. In addition, a recent Taiwanese study reported a strong association of the number of HZ related insurance claims with both UV and temperature [6].

In this study, we tested varicella and herpes zoster notification time series data for seasonality and correlation with UV and temperature. Previously, there were no studies investigating Australian VZV data in this context. As Australia is a vast and scarcely populated country with 8 climate zones, rather than attempting to analyse the nation-wide data we focused on a metropolitan location where population would be relatively large and UV would be generally high but substantially varying between seasons. The notification data collected in Perth, Western Australia (WA) were particularly suitable for this purpose. Perth has a large population by Australian standards—over 1.9 million in 2013 [7], concentrated in an area characterised by the same UV index. Perth has the most sunshine hours of the major Australian cities (8 to 9 hours daily) [8] and is located in a temperate climatic zone where the seasons are described in terms of European seasons applied to the southern hemisphere, so substantial variations in both temperature and UV take place every year [8, 9].

We note from the outset that notification data may not be an accurate representation of the actual incidence as they only represent the number of patients seeking medical attention. In addition, we acknowledge that a degree of case under-ascertainment and underreporting by doctors can be assumed. However, for the type of analyses we perform in this paper it is not crucial that the notification data should be as close to the real incidence as possible. We only need our data to behave qualitatively similarly to the real incidence in terms of seasonality. It is pertinent to mention that notification data are currently the only available kind of VZV data that can be used for systematic description of VZV dynamics in Australia. As there are usually no feasible mechanisms of collecting accurate VZV incidence data, VZV modelling studies commonly deal with some form of notifications such as insurance claims or dermatologist reports (see, for example, [5, 6, 10]), no matter that this may impose serious limitations on the results they obtain.

## Materials and Methods

### Data

We obtained the varicella and herpes zoster (shingles) notifications data collected in the Perth Metropolitan area from the Department of Health, Government of Western Australia. The data covered the period from January, 2009 to April, 2014 and were classified according to the terminology used by the National Notifiable Diseases Surveillance System (NNDSS): a confirmed case in this dataset was either based on laboratory definitive evidence and clinical evidence or clinical evidence and epidemiological evidence [11]. The data were stratified by sex and age (five-year age groups) and only included Perth residents. The age of individuals ranged from 0 to over 100 and there were 702 and 666 confirmed varicella cases, and 1,698 and 1,992 confirmed HZ cases in males and females, respectively. There were 2,237 and 2,591 cases marked as unspecified (i.e. VZV was confirmed but it was unclear whether it was varicella or HZ) in males and females, respectively. The number of notifications in our dataset suggests that the incidence of VZV in Perth may be low as compared with estimations for other countries [12].

**Fig 1:**
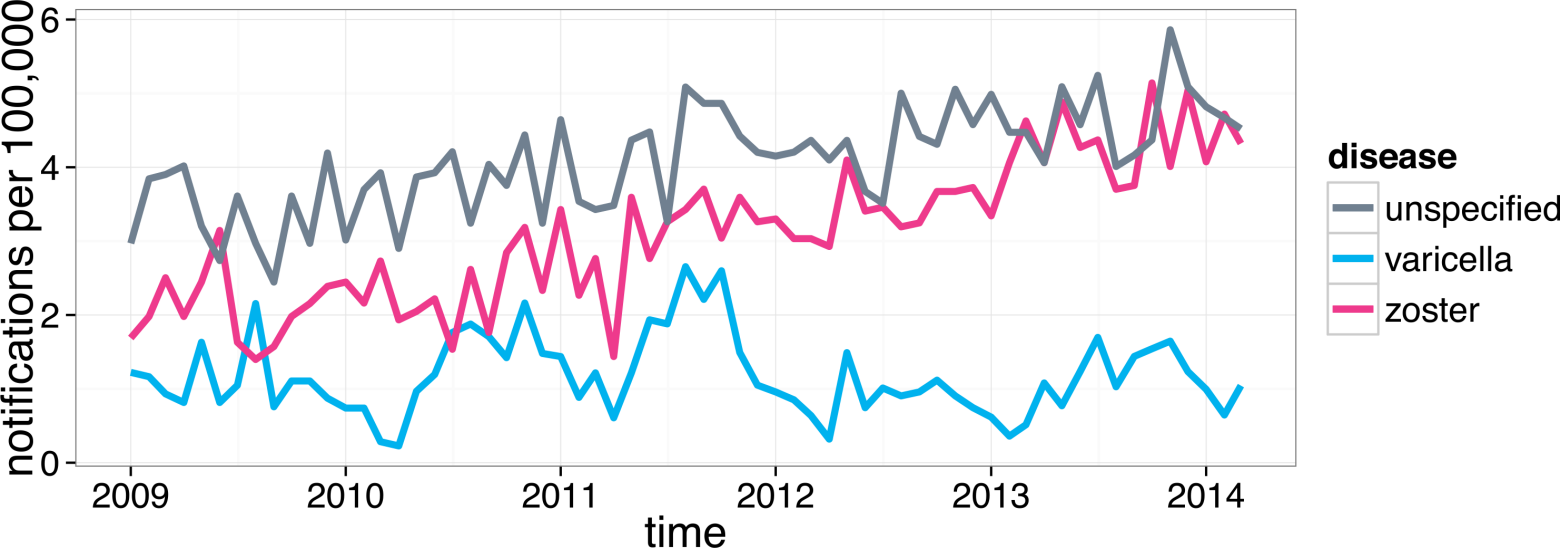
Monthly VZV notification rates in Perth Metropolitan Area (adjusted for the size of the at-risk population).

**Fig 2:**
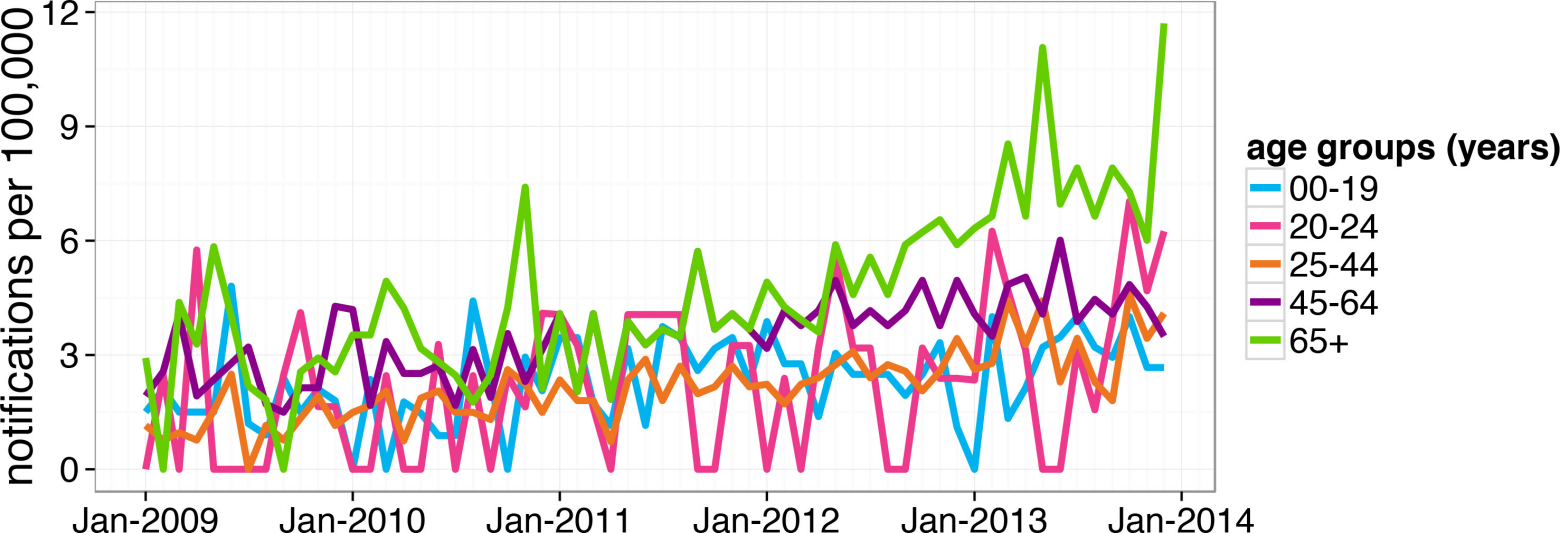
Monthly HZ notifications in Perth Metropolitan Area by age group (adjusted for variations in size of each group over years). We only included full years, so the data from the beginning of 2014 to April, 2014 are not shown. The groups are defined as in [14].

Each case record contained the optimal date of onset (ODOO). It is derived from the “true” date of onset of illness provided by the notifying doctor or obtained during case follow-up, or if this is not available the date of specimen collection for laboratory notified cases. Fig 1 shows the monthly VZV notification rates adjusted for the increase in the Perth population, from 2009 to 2014. Fig 2 shows the monthly HZ notification rates by age group, also adjusted for the changes in the number of individuals in each age group over the same period.

In this paper, notifications are used as the best available qualitative estimation of incidence. As we already mentioned in Introduction, we rely on the absence of dramatic variations in the quality of the NNDSS data in terms of VZV detection and reporting over the time period we consider. In 2004, which was 5 years before the time period we considered, an evaluation of NNDSS [13] revealed that the system was acceptably efficient and the data collected were generally matching quality expectations. Constant improvement of NNDSS since the time of evaluation should have made it very likely that any substantial disruptions in reporting or drops in quality of VZV detection would have been captured and reported by the system. While there is no publicly available evidence to indicate that the mentioned issues did not actually occur, Government of Western Australia has never voiced any concerns about reliability of the data in question.

It should also be noted that the notification data we used were collected after the introduction of varicella vaccination (publicly funded since November 2005 under the National Immunisation Program (NIP)). This is important in the context of a so-called progressive immunity hypothesis proposed by Hope-Simpson in the 60s [15], which states that (repeated) re-exposure to VZV boosts an individual’s level of cell-mediated immunity and consequently reduces the risk of HZ. The hypothesis is generally considered plausible although a number of studies have failed to identify any evidence to support it [1, 16].

The recorded maximum daily UV Index data for the period of interest was obtained from the Australian Radiation Protection and Nuclear Safety Agency (ARPANSA) [9]. The UV index is an international standard measurement of intensity of UV radiation reaching the surface of the earth [17].

In addition, we obtained the daily maximum air temperature data for Perth from the Bureau of Meteorology [18].

### Populations of Interest

We performed the analyses for the entire population as well as for certain subpopulations. In particular, we considered males and females separately, since there is evidence that epidemiology of VZV may be gender-specific to some extent, especially with regard to zoster [1, 19]. Age is clearly a crucial factor too, as HZ usually peaks at older age [1]. Hence, we divided individuals over 60 into additional gender-based groups of interest.

### Seasonality Analysis

As both climatic factors we considered (UV and temperature) tend to be distinctly seasonal, we began our investigation by testing the VZV time series for seasonality. It is appropriate to note that by season we mean a pattern in VZV notifications that increases and then decreases with some regularity (based on the definition given in [20]).

To investigate seasonality, we employed the methodology discussed in detail in [20] and implemented in an R package called “season” [21]. First, we adapted a simple approach known as the stationary cosinor model, which assumes that it is possible to adequately describe seasonal variations with a sinusoid. A cosinor model treats a response variable *Y*_*t*_ (which in our case is the notification counts at time *t*) as a linear combination of the sine and cosine functions with frequency *ω*_*t*_:

$$Y_{t}=c\cos(\omega_{t})+s\sin(\omega_{t})=A\cos(\omega t-P),$$ (1)

where $A=\sqrt{c^{2}+s^{2}}$ is the amplitude and *P* is a phase (the peak location). The model can be fitted as a generalized linear model (GLM).

A typical GLM is written as

$$g(E[Y_{t}])=\beta_{0}+\beta_{1t}X_{1t}+\ldots +\beta_{pt}X_{pt}$$ (2)

where *g*() is a link function that describes how the mean of the response *E*[*Y*_*t*_] and a linear combination of explanatory variables *X*_1_,*X*_2_,…,*X*_*p*_ (also known as predictors or independent variables) are related.

Next, we used the non-stationary parametric cosinor. This is a time series decomposition method using the Kalman filter [22] and Markov chain Monte Carlo sampling [23] that allows both amplitude and phase to be dependent on time. Decomposition here means that a time series is represented as a combination of the trend, season and error component. A full description of the methodology used in the non-stationary cosinor analysis is beyond the scope of this paper (see [20] for a detailed description).

Finally, we considered GLMs with months fitted as categorical independent variables to account for the possibility of non-sinusoidal seasonal patterns.

All computations were performed in the R environment for statistical computing [24].

### Interpretation of Unspecified Cases

Considering that the database we worked with contained a large number of unspecified cases (see Fig 1), we repeated our analysis under a number of assumptions regarding the proportions of unspecified cases representing varicella or HZ to determine how our results would be affected. The purpose of this was to verify if the reasonable expectation that nearly all cases in adults are HZ would translate into emergence or disappearance of seasonal patterns or change in the significance of any association of notifications with UV and/or temperature. Our base case assumption was that all unspecified cases in young children aged 0 to 9 were varicella, while all other unspecified cases were HZ. We considered variations of this assumption, assigning at least 70% of unspecified cases to HZ in individuals aged 10–14, at least 80% in those aged 15–19 and at least 90% in all older age groups. This analysis did not yield any results related to seasonality or association of notifications with UV and/or temperature that differed significantly from those obtained using the confirmed varicella and HZ cases.

## Results

### Seasonality using the cosinor models

The cosinor model [20] is suitable for describing simple seasonal patterns such as a sinusoid. This model only requires two parameters to produce a seasonal pattern (see Eq (1)) and is fitted as a part of a GLM. The cosinor model was fitted to varicella notifications and a statistically significant varicella annual seasonal pattern was detected based on an adjusted significance level of 0.025 (i.e. we would reject the null hypothesis if the p-value for either *s* or *c* from (1) was p<0.05/2). Varicella notifications peaked in August-September and were at their lowest in February-March. Both sine and cosine coefficients *s* and *c* were significant (p<0.001). The HZ notifications were clearly non-stationary (see Fig 1) and thus predictably not captured by the cosinor.

**Fig 3:**
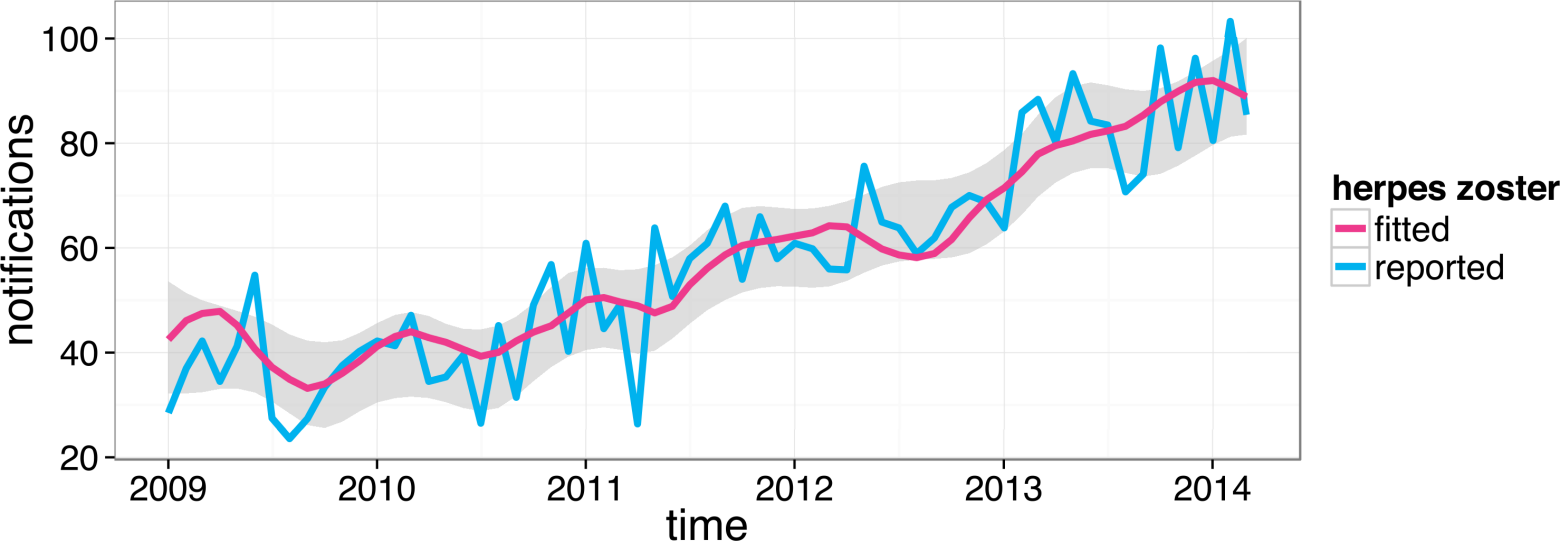
Non-stationary cosinor model fitted (red) to HZ notifications for Perth (blue). The fitted line is the mean and the grey area is the 95% confidence interval (both calculated based on 10,000 Markov chain Monte Carlo samples; see [20] for details).

Applying the non-stationary cosinor model we again observed strongly pronounced seasonality in varicella notifications. However, our main interest in using this model was to fit the HZ notification profile and extract a seasonal component from the HZ time series. The result of the fitting procedure is shown in Fig 3.

**Fig 4:**
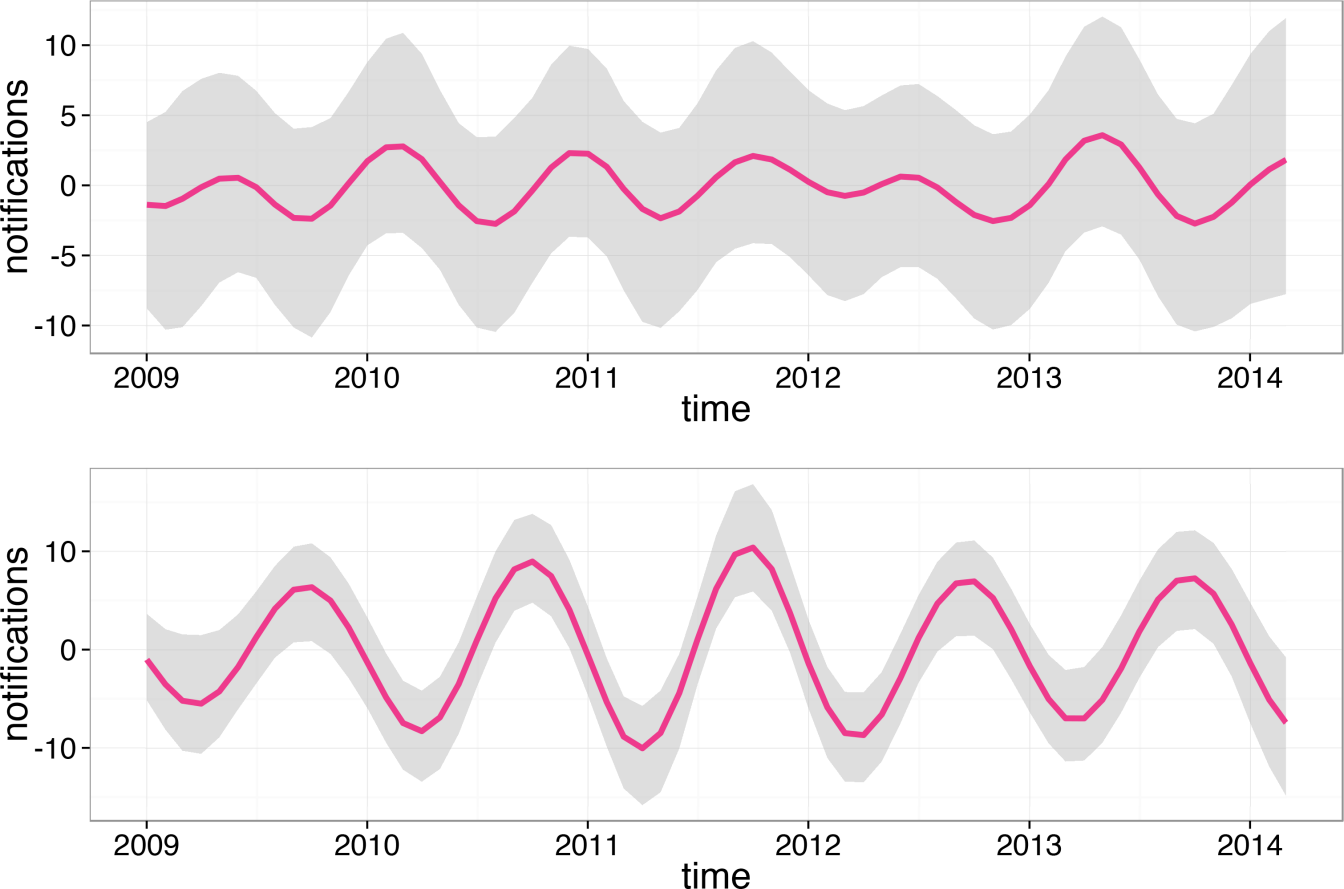
Seasonal component (mean over 10,000 iterations) for HZ (top) and varicella (bottom) in the entire resident Perth population. The grey area is the 95% confidence interval (calculated based on 10,000 Markov chain Monte Carlo samples, see [20] for details). Note that the seasonal component is conventionally centred to fluctuate around zero.

Note that monthly counts were adjusted for the size of the at-risk population and the number of days per month. The former adjustment is particularly important because the Perth population has been rapidly growing since 2009. For example, between 2013 and 2014 it increased by 2.5% [7]. The seasonal component did not follow any apparent annual pattern as can be seen in Fig 4.

The seasonal component of the varicella time series is also shown in Fig 4. Note that the mean phase for varicella was around August, in agreement with the fit produced by the stationary cosinor.

**Fig 5:**
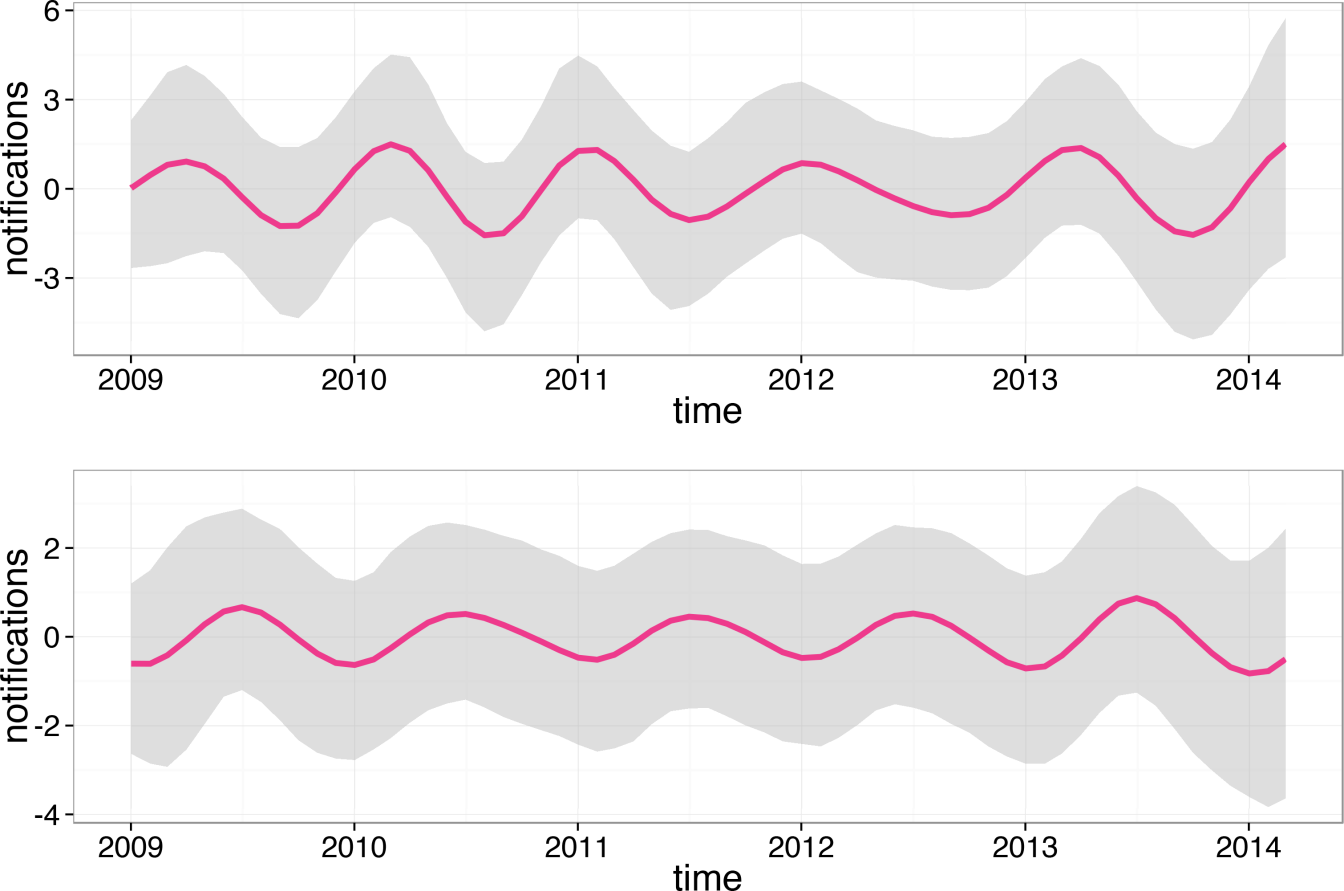
Seasonal component for HZ in the 60+ Perth females (top) and males (bottom). The grey area is the 95% confidence interval (calculated based on 10,000 Markov chain Monte Carlo samples, see [20] for details).

When we fitted the HZ notifications for males and females separately we observed that in females the seasonal component was weak but it did show consistent amplitude until 2012 when it started to flatten. On the contrary, in males the amplitude was hardly noticeable until 2011 when it became clearly pronounced and showed signs of further growth in early 2014. We also fitted the HZ notifications for males and females over 60 (together and separately) as they are at a high risk of HZ due to older age. Seasonal components for the 60+ groups are shown in Fig 5.

For 60+ females we observed a varying phase and a temporarily flattening sinusoid in 2012 while in 60+ males the sinusoid had roughly the same amplitude each year peaking consistently around June.

### GLM for HZ Notifications with Months as Categorical Variables

In order to assess the association of climatic factors we considered a GLM with months included as categorical variables. Specifically, our model was of the form

$$g(E[Y_{t}])=\beta_{0}+\beta_{1t}X_{1t}+\beta_{2t}X_{2t}+\beta_{12t}(X_{1t}\circ X_{2t})+[months]$$ (3)

where the response *Y* was the HZ counts assumed to be Poisson distributed, *X*_1_ was the temperature, *X*_2_ was the UV, and the term *X*_1_ ∘ *X*_2_ corresponded to their interaction. We utilised a *g*() = log() link function typically used for counts. Implementation of the model was based on a modification of the standard R function glm() called monthglm() available in the R package “season” [21].

For the entire population, all months but February were significant (p<0.05) as were temperature and UV (the corresponding rate ratios (RRs) for temperature and UV were 1.21 and 2.01, respectively). Recall that for an increase in an explanatory variable by 1, a percentage change in the dependent variable is (*RR*−1)×100% so RR = 1.21 for temperature means that a 1 degree increase in temperature is associated with a 21% increase in. Similarly, RR = 2.01 for UV means that an increase of 1 in the UV index is associated with a 101% increase in HZ notifications. The interaction between temperature and UV was also significant (RR = 0.98, p<0.05).

For males, the model (Eq (3)) was modified to have the HZ count in males only as a response with other variables remaining intact. All months except for December were significant, as were temperature (RR = 1.16, p<0.04) and UV (RR = 2.07, p<0.007). For males over 60, we observed a similar outcome except for temperature was insignificant. For all females and for females over 60 both temperature and UV were significant (RR at least 1.2 and p not exceeding 0.008).

## Discussion

In this paper we analysed a VZV notification time series from Perth, Western Australia. The analysis was focused on an assessment of seasonality and on determining the association of the amount of UV radiation reaching the surface of the earth (measured by the UV index recorded in Perth) and temperature with VZV notification dynamics.

### Descriptive Epidemiology

The low number of varicella notifications for Perth as compared with many other countries [12] is likely to be due to an extensive varicella vaccination (it is estimated that 88.8% of children under 24 months of age had been immunised in Western Australia by the end of 2014 [25]). The relatively low number of HZ notifications (only 3,690 confirmed cases in a population of about 1.9 million during the period of interest) does not necessarily imply an abnormally low level of HZ in Perth because this number would be roughly doubled if we assigned all or nearly all unspecified VZV cases in adults to HZ. Importantly, we verified that doing so would not alter our results. Even lower numbers of HZ notifications have been previously reported for some cities (see for example [26] where 1,798 HZ cases over 8 years were documented for Madrid whose population exceeds 3 million).

The HZ notification rate has been increasing in Perth since 2009, particularly in older age groups (Fig 2). This is consistent with the trends reported to be taking place in a number of other countries [27] and generally in Australia [28, 29]. At the same time, varicella notifications have been declining and the ratio of HZ notifications to varicella notifications has been increasing (Fig 1). Vaccination is a possible cause of this effect [27]. The Hope-Simpson hypothesis [15] implies that an extensive varicella vaccination would drastically reduce VZV incidence in children and consequently exposure to VZV in adults, which then would result in increasing HZ incidence. Note that the time period shown in Fig 1 starts 4 years after publicly funded varicella vaccination commenced in Australia [25].

While vaccination is arguably the most convincing explanation for increasing HZ notifications, in some countries an increase in HZ was noted before the introduction of varicella vaccination programmes [6, 12]. In addition, results from the only study to date that examined trends in HZ notifications following the introduction of universal funding for varicella immunization in Australia indicated that it was not possible to directly attribute the increasing trends in observed HZ notifications to the immunization programme [28].

### Seasonality of Varicella

**Fig 6:**
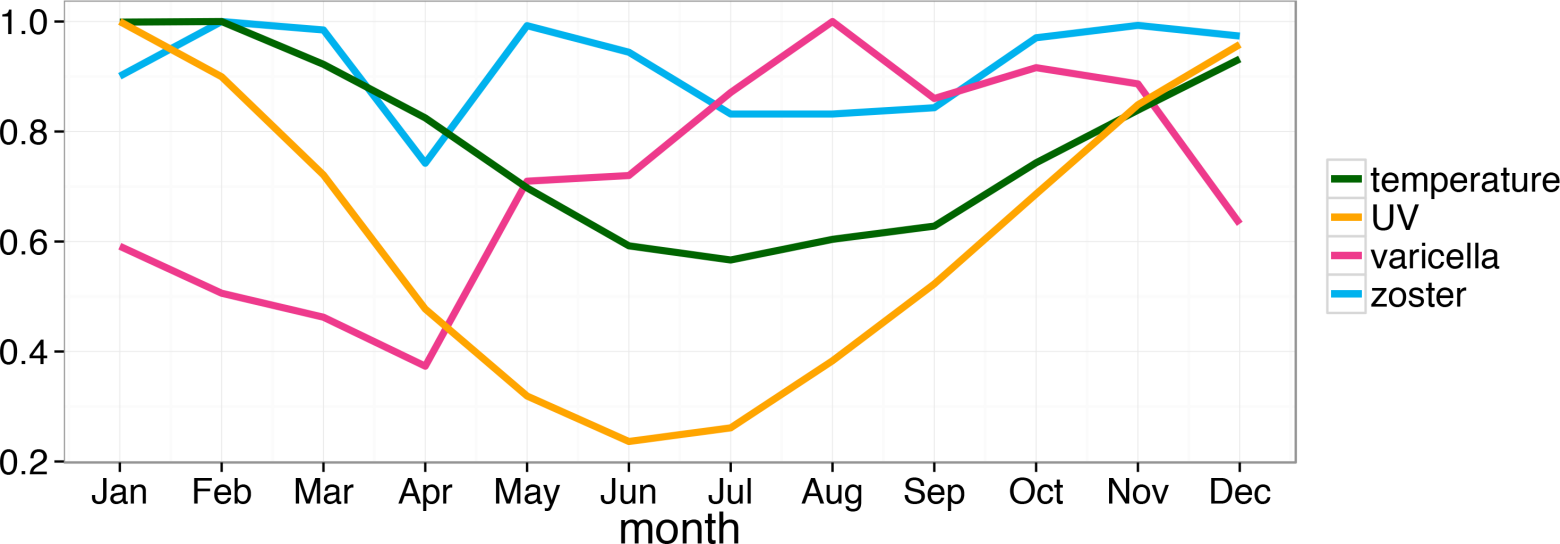
Normalised varicella and HZ notifications for the resident Perth population, maximum UV and temperature averaged by month over the period of January, 2009 to April, 2014.

Previous studies have suggested that varicella incidence can exhibit seasonal patterns, particularly in temperate climates [6], however in only a few cases it was strongly pronounced [10, 19]. We identified significant seasonality of varicella notifications that is well described by both stationary and non-stationary sinusoids. Previously, seasonality was detected in varicella data from Victoria and South Australia using Edward’s test [30] but it was concluded that a sinusoid could be unsuitable to describe it. In our analysis of varicella notifications for Perth we identified seasonality with the largest number of notifications typically observed in August-September. This was not the case in the aforementioned analyses of notification data for Victoria and South Australia, where varicella tended to peak in summer months (December-February). Like Perth, Victoria and South Australia are predominantly in the temperate climate zone. As mentioned in [31], several studies have found the highest incidence of varicella to occur in the coolest months of the year (see, for example, [32]), which is not dissimilar to what we observe for Perth where the coolest period lasts from June to August (winter), with July being the coldest month on average (see Fig 6).

### Seasonality of Herpes Zoster

Nearly all studies conducted in Australia or elsewhere that have attempted to detect seasonality of HZ were unsuccessful in this matter [1]. To our knowledge there are two exceptions: one study conducted in Lodz, Poland showed that there was significant seasonality of zoster notifications for open body sites in males but not in females in the period 1999–2000 [5]; a more recent and much more extensive Japanese and Taiwanese studies [6, 10] demonstrated seasonality of both varicella and HZ notifications that mirrored each other [10], suggesting that when varicella was increasing HZ was declining and vice versa. Note that we did not see this mirroring effect consistently showing in Perth (see Fig 1 and Fig 4).

An Australian study that addressed the question of HZ seasonality using sinusoidal models [30] failed to detect it, although an increase in HZ-specific antiviral prescriptions during summer months was reported [28]. The National GP Disease Surveillance Network data for 1994–1995 also showed no seasonal variation in rates for HZ [30]. We should also mention that an extensive Bettering the Evaluation and Care of Health (BEACH) national cross-survey dataset containing HZ management rates was analysed in [29] but seasonality was not among the questions addressed in the study.

The HZ notification time series decomposed using the non-stationary cosinor model showed a weakly pronounced seasonal component for the studied time period. The phase of the seasonal component varied between years and was only stable in older males. This suggests that the non-stationary cosinor model may be inadequate for capturing HZ seasonality or that the seasonality was non-existent in all subpopulations but older males. Another possibility is that there was a category of individuals not explicitly represented in our data that was contributing a seasonal component to otherwise non-seasonal HZ profiles for the subpopulations we considered. For example, this category might be comprised of individuals with HZ on open body sites as in [5]. It is also possible that seasonality of HZ was affected by vaccination and the subsequent drop in varicella incidence in children but our current knowledge of Australian age-specific contact patterns (see loose estimations provided in [14]) is not sufficient to evaluate this possibility with any certainty. Vaccination may also explain the differences in phase for varicella notifications between Perth and two other Australian states (Victoria and South Australia) as the data analysed in [30] were collected before vaccination commenced.

### Association of HZ with Climatic Factors

Our results in relation to seasonality, obtained using the non-stationary cosinor, did not provide strong indications that HZ notifications would be associated with climatic conditions, but further GLM analysis revealed a significant association with UV for all considered population groups. In [6], it was found that HZ notification data exhibited a strong association with UV and temperature for the Taiwanese population and we also observed significant association with temperature in most cases, although it was usually less significant than for UV. This was also true for the populations of older males and females. They are likely to be less immune than younger individuals but just as exposed to UV. The latter is clearly supported by behavioural data describing usage of outdoor sports facilities, parks or reserves and public playing fields or ovals [33] as well as data on how Australians usually spend their time [34]: older individuals certainly do not tend to stay indoors much more than younger people. For example, over 45% of males over 65 attend parks, which is the highest proportion among males of any age. Hence, the older Perth residents are unlikely to be less exposed to UV due to their lifestyle.

### Limitations

Out study has two important limitations. First of all, there was no reliable way to ascertain how closely the notification dataset we used reflected the actual varicella and HZ incidence. There could be variations in quality of detection and notification that affected the recorded notification counts. Specifically, there could be changes in testing and notification by doctors as well as availability and use of more sensitive polymerase chain reaction tests. In addition, some individuals with milder cases of varicella and zoster may not seek medical attention, so these may remain unreported.

As detailed in Introduction, many VZV modeling studies face similar limitations as the actual VZV incidence data are usually unavailable (see, for example, [5], [6] or [10]). In view of this, our efforts to verify the robustness of our results by re-applying analysis to the original data augmented via re-categorizations of the unidentified VZV notifications gave us an additional degree of confidence regarding suitability of our data for the purpose of the study.

Secondly, we had no suitable data to properly assess the actual UV exposure for various categories of individuals. While the recorded UV index values inform about the UV level at the surface of the earth, behavioral data are needed to understand how these are correlated with the amount of time people spend in the sun and related factors such as the use of sunscreen. Whereas convincing biological explanations of the observed differences in VZV between males and females are currently absent, we can only presume that the apparently higher HZ incidence in females may be due to some differences in behavior, which is an assumption similar to that made in [5]. There are data suggesting that Australian males tend to spend more time outdoors than females [34] and still males appear to be less prone to HZ. This emphasizes that even though we established a significant association of HZ notifications with UV, we do not have an understanding of what mechanism underlies this observation.

### Summary

In conclusion, we confirmed seasonality of varicella in Perth, Western Australia, which peaked during August-September (Australian spring when both UV and temperature are relatively low). The HZ seasonal component was most consistent between years in older males, peaking in around June (one of the coldest months in Australia). Significant association between HZ notifications and UV for the Perth resident population was established and in most cases, temperature proved to be a significant factor as well. These findings may contribute to our understanding of the importance of UV in VZV epidemiology. High quality behavioural data clearly describing the actual unprotected exposure to UV are needed in order to further clarify the effect of UV and temperature on VZV.
